# A review of Phase I trials of Ebola virus vaccines: what can we learn from the race to develop novel vaccines?

**DOI:** 10.1098/rstb.2016.0295

**Published:** 2017-04-10

**Authors:** Teresa Lambe, Georgina Bowyer, Katie J Ewer

**Affiliations:** The Jenner Institute, University of Oxford, Old Road Campus Research Building, Roosevelt Drive, Headington, Oxford OX3 7DQ, UK

**Keywords:** Ebola virus, vaccine, clinical trial, T cell, antibody, emerging pathogens

## Abstract

Sporadic outbreaks of Ebola virus infection have been documented since the mid-Seventies and viral exposure can lead to lethal haemorrhagic fever with case fatalities as high as 90%. There is now a comprehensive body of data from both ongoing and completed clinical trials assessing various vaccine strategies, which were rapidly advanced through clinical trials in response to the 2013–2016 Ebola virus disease (EVD) public health emergency. Careful consideration of immunogenicity post vaccination is essential but has been somewhat stifled because of the wide array of immunological assays and outputs that have been used in the numerous clinical trials. We discuss here the different aspects of the immune assays currently used in the Phase I clinical trials for Ebola virus vaccines, and draw comparisons across the immune outputs where possible; various trials have examined both cellular and humoral immunity in European and African cohorts. Assessment of the safety data, the immunological outputs and the ease of field deployment for the various vaccine modalities will help both the scientific community and policy-makers prioritize and potentially license vaccine candidates. If this can be achieved, the next outbreak of Ebola virus, or other emerging pathogen, can be more readily contained and will not have such widespread and devastating consequences.

This article is part of the themed issue ‘The 2013–2016 West African Ebola epidemic: data, decision-making and disease control’.

## 1. Introduction

The 2013–2016 epidemic of Ebola virus disease (EVD), in West Africa, was unprecedented in both scale and spread. The case count and death toll has surpassed the total number of cases for all previous known outbreaks. This epidemic has caused more than 11 300 deaths with more than 28 600 cases: figures widely thought to be underestimated [1]. Development and deployment of efficacious EVD therapeutics and vaccines could have played a more significant role in limiting the 2013–2016 outbreak and remain a priority for the prevention of future EVD epidemics [2].

The family *Filoviridae* includes Marburg and five *Ebolaviruses*, named for their location of recognition: Zaire (now known as Ebola virus, EBOV), Sudan (SUDV), Reston (RESTV), Taï Forest (TAFV) and Bundibugyo virus (BDBV) [3]. The single protein expressed on the surface of filoviruses, the glycoprotein (GP), is antigenically and immunologically important and is frequently therapeutically targeted. However, bioinformatics analysis, at the structure-based and amino acid level, demarcates limited regions of homology in GP [4] and also large areas of divergence particularly in the head domain of GP. It is therefore unclear if exposure to one member of the family *Filoviridae* confers cross-protective immunity.

It is unclear if the immune response involved in Ebola virus clearance is dependent on a single aspect of the immune system (cellular or humoral immunity) or if a multifaceted response is a prerequisite and at present there is no clear correlate of protection for EVD. Indications from experimental infection of vaccinated non-human primates suggest a role for GP-specific IgG and CD8^+^ T cells [5,6], and the limited analyses of convalescent samples suggest that the presence of Ebola virus-specific IgG and intact cell-mediated immunity (CMI) are associated with survival from natural infection [7–9]. These observations are supported by immunological analysis of survivors from the current outbreak, which indicates a multi-faceted response is induced post-infection with strong humoral immunity and significantly pronounced transcriptional changes in CD8^+^ T cells in response to several Ebola virus proteins [10].

EVD vaccine development has been well established since the discovery of Ebola virus and a number of different vaccine platforms have been used, resulting in at least one efficacious vaccine. These vaccine platforms include DNA, recombinant or subunit proteins, virus-like particles (VLPs) and recombinant viral vectors. A number of these vaccines have advanced past pre-clinical testing and are undergoing clinical testing; generally, those modalities that have demonstrated efficacy or high levels of immunogenicity in pre-clinical models. The most clinically advanced vaccines against EVD are based on generating immune responses toward GP with trials ongoing in Europe, the USA and Africa. There are eight vaccines in clinical trials, all targeting the Ebola virus GP, with some regimens employing a single dose and others using two vaccines in a heterologous prime-boost approach. Prime-boost regimens have been shown to be more immunogenic than single-dose vaccinations for diseases such as malaria; however, there are financial and logistical implications associated with administering two vaccines [11]. The Ebola virus vaccines that are currently being assessed differ in the qualitative immune response post-vaccination, which may be due to the use of alternate vaccine platforms. Indeed, while humoral immunity may be critical for protection post vesicular stomatitis virus (VSV)-vectored vaccines in non-human primates (NHPs), CMI may be key post single-shot adenovirus vectored vaccines [12]. As both humoral and cellular immunity have been demonstrated to be protective in NHP, and are induced in man post-EBOV infection, a vaccine regimen which induces long-lived and sustainable levels of CMI and antibodies is desirable. Primarily, and finally, only those vaccines that are safe, efficacious and deployable will be field-effective, therefore only vaccine candidates that have progressed to published Phase I studies will be reviewed here. Clinical trials where results were published in peer-reviewed journals by July 2016 are summarized in table 1.

**Table 1.** Summary of Phase I clinical trials of vaccines for EVD. vp, viral particles; pfu, plaque-forming units.

vaccine type	vaccine(s)	clinical trials.gov identifier	vaccination start month	dose(s) used	regimen	site	*N*	references
DNA vaccine	VRC-EBODNA012-00-VP-DNA: 3-plasmid (transmembrane-deleted EBOV GP, SUDV GP, nucleoprotein)	NCT00072605	November 2003	2, 4 or 8 mg	three doses at weeks 0, 4, 8	MD, USA	27 (5, 8, 8 + 6 placebos)	[13]
replication-deficient viral vectors	rAdHu5 EBOV and SUDV GP with single point mutation in GP (asp- > glu at position 71)	NCT00374309	September 2006	2 × 10^9^ vp, 2 × 10^10^ vp	single dose	MD, USA	31 (12, 11 + 8 placebo)	[14]
DNA vaccine	VRC-EBODNA023-00-VP 2 plasmids—SUDV and EBOV GPs with a Marburg DNA vaccine (full-length WT GP)	NCT00605514	January 2008	4 mg	three doses at weeks 0, 4, 8, fourth dose boost at 32 weeks	MD, USA	20	[15]
DNA vaccine	VRC-EBODNA023-00-VP 2 plasmids—SUDV and EBOV GPs with a Marburg DNA vaccine (full-length WT GP)	NCT00997607	November 2009	4 mg	three doses at weeks 0, 4, 8	Kampala, Uganda	30 EBOV vaccine, 30 Marburg vaccine, 30 both concomitantly	[16]
replication-deficient viral vectors	mixture of ChAd3 EBOV and SUDV GP	NCT02231866	September 2014	1 × 10^10^ vp, 1 × 10^11^ vp	single dose	MD, USA	20 (10 per dose)	[17]
replication-deficient viral vector	ChAd3 EBOV GP	NCT02240875	September 2014	1 × 10^10^ vp, 2.5 × 10^10^ vp, 5 × 10^10^ vp	single dose	Oxford, UK	60 (20 per dose)	[18]
replication-deficient viral vectors	ChAd3 EBOV GP and MVA-BN Filo	NCT02240875	September 2014	ChAd3 at: 1 × 10^10^ vp, 2.5 × 10^10^ vp, 5 × 10^10^ vp MVA at: 1.5 × 10^8^, 3 × 10^8^ pfu	ChAd3 at D0, MVA at 3–10 weeks, 1 week or 2 weeks	Oxford, UK	30 (15 each dose), split across the ChAd3 dose groups	[18]
live replicating viral vaccine	rVSV-ZEBOV	NCT02269423 NCT02280408	October 2014	3 × 10^6^, 2 × 10^7^ pfu	single dose	Washington DC, Baltimore MD, USA	52 (26 at each site, 10 + 3 placebos in each dose group)	[19]
replication-deficient viral vector	ChAd3 EBOV GP	NCT02289027	October 2014	2.5 × 10^10^ vp, 5 × 10^10^ vp	single dose	Lausanne, Switzerland	120 (100 vaccinees, 20 placebo)	[18,20]
replication-deficient viral vector	ChAd3 EBOV GP	NCT02267109	October 2014	1 × 10^10^ vp, 2.5 × 10^10^ vp, 5 × 10^10^ vp, 1 × 10^11^ vp	ChAd3 at D0, MVA at 11–16 weeks	Bamako, Mali	91	[21]
live replicating viral vaccine	rVSV-ZEBOV	NCT02296983 NCT02283099 NCT02287480	November 2014	3 × 10^5^, 3 × 10^6^, 1 × 10^7^, 2 × 10^7^, 5 × 10^7^ pfu	single dose	Kilifi, Kenya; Hamburg, Germany; Geneva, Switzerland	158	[22]
replication-deficient viral vectors	AdHu26 EBOV GP and MVA-BN Filo	NCT02313077	December 2014	ChAd3 at 5 × 10^10^ vp MVA at 1 × 10^8^	Chad3 or MVA at D0, heterologous boosting at 2, 4 or 8 weeks	Oxford, UK	87 (75 vaccinees and 12 placebo)	[23]
replication-deficient viral vector	rAdHu5 encoding EBOV GP from 2014 outbreak strain	NCT02326194	December 2014	4 × 10^10^ vp, 1.6 × 10^11^ vp	single dose	Jiangsu, China	120 (40 low dose, 40 high dose, 40 placebo)	[24]
live replicating viral vaccine	rVSV-ZEBOV	NCT02287480	January 2015	3 × 10^5^ pfu	single dose	Geneva, Switzerland	56 (51 vaccinees and 5 placebo)	[25]

## 2. Safety and reactogenicity of vaccines against *Ebolavirus* disease

The earliest clinical studies of vaccines against EVD used plasmid DNA encoding the nucleoprotein from the *Zaire ebolavirus* and the glycoproteins from EBOV and SUDV [13,15]. Although well tolerated with mostly mild and short-lived adverse events (AEs), DNA vaccines tend to be poorly immunogenic and have been largely superceded by subunit proteins, recombinant viral vectors and VLPs in several vaccine development fields, including Ebola virus [16]. The vaccines most progressed for the prevention of EVD use viral vector platform technology and use either the adenovirus, modified vaccinia virus Ankara or vesicular stomatitis virus backbones.

Recombinant human serotype 5 adenovirus (AdHu5) vectors encoding GP have been evaluated in single-dose studies and have shown very acceptable safety profiles. Unfortunately, AdHu5-seropositive volunteers demonstrated statistically significantly lower GP-specific IgG titres [14]. Increasing the administered dose of vaccine appears to overcome pre-existing immunity to this serotype; however, this is associated with increased reactogenicity [24]. Unfounded concerns also still persist about a potential increase in HIV-1 infection rates among AdHu5-seropositive vaccinees, based on data from two Phase II studies of the Merck rAd5 HIV-1 gag/pol/nef vaccine (the STEP and Phambili trials) [26–28]. Use of rarer serotypes of human adenoviruses, such as Ad26 and AdHu35, can bypass pre-existing immunity [29] and have demonstrated acceptable safety and tolerability profiles.

The most widely evaluated adenoviral vector EVD vaccines are based on a simian adenovirus serotype 3 backbone (ChAd3) encoding either *Ebolavirus* glycoprotein alone [18,20] and administered as a single shot or as a mixture of ChAd3 EBOV and ChAd3 SUDV vaccines [17]. Between September 2014 and January 2016, four Phase I studies of these vaccines were undertaken, one each in the US, UK, Switzerland and Mali. Replication-deficient chimpanzee adenovirus vectors have been extensively evaluated as candidate vaccines for malaria, HIV, HCV, RSV, influenza and tuberculosis, in adults, children and infants, with no safety concerns [30]. Safety profiles were similar for candidate Ebola virus vaccines, with higher doses eliciting more AEs; however, AEs were mostly graded as mild, resolving within 24 h of vaccination.

An additional vaccine modality being tested is modified vaccinia Ankara (MVA); both unmodified and recombinant MVA vectored vaccines have been evaluated extensively as vectors for smallpox, malaria, flu and TB vaccines with excellent safety demonstrated in immunocompetent and HIV-infected participants [31,32]. In pre-clinical trials, ChAd3 was observed to provide 100% protection when NHPs were challenged with Ebola virus shortly after vaccination; however, an MVA boost was required to enable uniform protection when challenge occurred 10 months after priming vaccination. This increased durability of protection was attributed to the superior effector and memory CD8^+^ T cell responses elicited by the MVA boost [5]. Owing to the superior durability of protection demonstrated by the prime-boost regimen in pre-clinical trials, several Phase I clinical trials were initiated to test this regimen in humans. MVA-BN Filo is a recombinant, replication-deficient MVA vector, encoding EBOV, SUDV and Marburg glycoproteins as well as TAFV nucleoprotein. Two clinical trials have assessed a ChAd3 prime with MVA-BN Filo boost, demonstrating acceptable safety profiles and significantly enhanced cellular and humoral immunogenicity compared with a single-shot ChAd3 vaccination [18,21].

The other vaccine modality being assessed as an Ebola vaccine candidate is the recombinant vesicular stomatitis virus encoding EBOV glycoprotein (rVSV-ZEBOV), which is an attenuated replication-competent viral vector. Unmodified VSV causes either asymptomatic infection or a mild, flu-like illness in humans [33]; however, truncation of the VSV glycoprotein results in an attenuated, avirulent form that has been shown to be safe in pre-clinical studies in macaques and mice with no evidence of viral shedding [34]. A rVSV HIV-1 *gag* vaccine has also generated acceptable safety data in two Phase I studies [35,36]. Candidate vaccines using these vectors also elicited protection against both Ebola and Marburg viruses in a NHP model where several rVSV each encoding a single filovirus glycoprotein were mixed and injected in a single dose that elicited protection against a lethal challenge [37]. Six Phase I studies of rVSV-ZEBOV were initially performed between October 2014 and January 2015, two in the US and one each in Kenya, Gabon, Switzerland and Germany [19,22]. Almost all participants displayed transient rVSV viraemia, starting at day 1, and lasting beyond day 7 in around 10% of participants, with mostly mild and moderate AEs lasting up to 24 h on average. In total, 22% (38/170) of participants across the six trials had documented fever after vaccination, significantly higher than the 8% observed after vaccination with ChAd3 (15/190, *p* = 0.0002, two-tailed Fisher's exact (Prism version 6, GraphPad Software)). Although both vaccines have been evaluated across a range of doses, post-immunization fever levels are an important consideration for a vaccine that may be deployed in an outbreak of a highly febrile illness, such as EVD. In the Geneva trial, 11 of 51 participants developed arthritis lasting a median of 8 days, with virus subsequently identified in synovial joint fluid, indicating peripheral viral replication; two cases of shorter duration were identified among participants in the other studies. A further study in Geneva where a much lower dose of rVSV-ZEBOV was evaluated in a further 51 volunteers resulted in much lower total IgG and neutralizing antibody titres and was again associated with arthritis in 25% of participants [25]. Transient arthritis is often observed after immunization with commonly used live vaccines, such as rubella [38]; however, when rVSV-ZEBOV progressed to a large-scale Phase III cluster-randomized efficacy trial, this particular adverse event was not reported and, in addition, in an interim analysis the vaccine elicited high levels of efficacy in EVD-exposed participants [39]. Incidence of post-vaccination fever in that study has not yet been reported.

In summary, all of the candidate vaccines for EVD that progressed to Phase I studies in response to the 2013–2016 outbreak demonstrated acceptable safety profiles; therefore, describing the magnitude and characteristics of vaccine-induced immunity was the next scientific aim.

## 3. Humoral immune response to *Ebolavirus* vaccines

In the absence of any gold standard for measuring humoral immunity against Ebola virus, a wide variety of assays were used to evaluate antibody responses to vaccines during the recent EVD outbreak. Binding assays were used to quantify antibody binding to recombinant protein or inactivated whole virion while neutralization assays were used to assess the functional capacity of antibodies.

### (a) Binding assays

Binding assays, particularly enzyme-linked immunosorbent assays (ELISAs), are commonly used to assess the quantity of antigen-specific antibody induced post-vaccination and can additionally be used to measure qualitative aspects of the humoral immune response such as isotype profiles and *in vitro* avidity.

*Ebolavirus* outbreaks occur sporadically and have previously been considered too limited in size to enable assessment of vaccine efficacy prior to regulatory approval. In 2002, the USA Food and Drug Administration (FDA) introduced the ‘animal rule’ enabling data from pre-clinical trials to be used to demonstrate efficacy when human trials were not possible as an alternative licensing route for drugs and vaccines against highly lethal diseases [40].

Despite the wide use of ELISA assays to measure anti-glycoprotein IgG responses, the majority of Phase I studies used an array of different antibody binding assays to assess the quantity and quality of the antigen-specific antibody response induced by vaccination (table 2). The use of different assays conducted in different laboratories following different protocols hinders the comparison of humoral immunogenicity induced by different vaccine candidates. Key differences in these assays that affect comparability of results include the use of glycoprotein from different strains of Zaire Ebola virus (Kikwit, Makona or Mayinga), use of whole virion or recombinant protein and the use of different read-outs or units. Centralized standard assays or readily available biological standards for a range of emerging pathogens would be useful tools to aid decision makers in choosing the most promising candidate to take forward for further testing and rapid deployment during outbreaks. A WHO reference reagent for anti-EBOV IgG has been established by the WHO Expert Committee on Biological Standardisation (ECBS) for use as a reference standard in humoral immunoassays including neutralization and ELISAs against Ebola virus [41]. It is now freely available from the National Institute for Biological Standards and Control (NIBSC, UK) and should facilitate retrospective comparison of responses between different trials.

**Table 2.** Assays used to evaluate humoral responses to Ebola vaccine candidates tested during the 2013–2016 outbreak.

type	assay	protein/target	Ebola strain	parameter measured	read-out	references
binding assays	Ebola GP standardized ELISA	EBOV GP (recombinant)	Mayinga	total IgG binding to recombinant EBOV GP	arbitrary ELISA units	[18]
	whole virion ELISA	EBOV	Makona	total IgG binding to inactivated EBOV virion	arbitrary ELISA units	[18,22,25]
	Alpha Diagnostics (ADI) kit ELISA—Sudan Ebola virus (AE321620-1)	SUDV GP (recombinant)	unknown	total IgG binding to recombinant EBOV GP	OD450	[18]
	Alpha Diagnostics (ADI) kit ELISA—Zaire Ebola virus (AE320620-1)	EBOV GP (recombinant)	unknown	total IgG binding to recombinant EBOV GP	OD450^a^	[18,20,25]
	‘NIH’ ELISA	EBOV GP (recombinant)	Mayinga	total IgG binding to recombinant EBOV GP	titres calculated from an EC90 value	[16–21]
	‘NIH’ ELISA	SUDV GP (recombinant)	Gulu	total IgG binding to recombinant SUDV GP	titres calculated from an EC90 value	[16,17]
	Ebola virus competition ELISA	EBOV GP (recombinant)	Mayinga	EBOV GP-specific IgG able to displace an Ebola-neutralizing monoclonal antibody MAb 4G7	% reduction in HRP-conjugated MAb binding	[18]
	US Army MRIID anti-GP ELISA	EBOV GP (recombinant)	Kikwit	total IgG binding to recombinant EBOV GP	endpoint titre	[19,22,25]
functional assays	live virus neutralization	live Ebola virus	Mayinga	capacity of antibody to neutralize live Ebola virus *in vitro*	neutralizing titre	[18,22]
	pseudotyped lentivirus neutralization	pseudotyped lentivirus expressing EBOV GP	Mayinga	capacity of antibody to neutralize a lentivirus expressing EBOV GP	IC50	[18]
	pseudotyped VSV ELISA	VSV pseudovirus expressing EBOV GP	Kikwit	capacity of antibody to neutralize VSV expressing EBOV GP	endpoint titre	[19,22,25]

^a^DeSantis *et al.* [20] converted OD450 into estimated μg ml^−1^ using positive control included in the kit and 1 : 200 sample dilutions. Ewer *et al.* [18] used 1 : 500 dilutions.

To aid in the comparison of immunogenicity across trials, samples from a recent Phase I trial of ChAd3 MVA-BN Filo [18] were run on a large number of these assays and correlation analyses were conducted (table 3). A lack of correlation between some of these assays may indicate that they measure different aspects of the humoral immune response and may therefore correlate differently with protection. For example, the competition ELISA used by Ewer and co-workers did not correlate with neutralizing titre to live Ebola virus Mayinga strain or several of the glycoprotein ELISAs. The competition ELISA measures the ability of anti-EBOV antibodies in a sample to compete with the monoclonal EBOV antibody (mAb) 4G7 and therefore only detects activity against a single GP epitope. The lack of a correlation with neutralizing titre may indicate that antibodies binding to this epitope are non-neutralizing and that the protection that has been observed when this mAb has been used in a mAb cocktail in NHP models is conferred by an alternative mechanism [42]. Many of the other binding assays used in these Phase I trials correlate strongly and, in particular, it is useful that the standardized glycoprotein ELISA and pseudotyped lentivirus assays each correlate strongly with neutralizing titres against live Ebola virus as these assays avoid the need to work at high containment levels, but may be used to indicate the presence of antibodies with neutralizing activity.

**Table 3.** Relationships between different assays used to assess humoral responses to Ebola vaccine candidates. Spearman's rank and *p*-values for correlations between each of the assays tested. The same 30 samples were run on each assay. Samples were serum from healthy UK volunteers in a Phase I trial of ChAd3_MVA EBO Z conducted at the University of Oxford [18]. All samples were from the same time point—two weeks after MVA boost. ADI ELISA, pseudotyped lentivirus neutralization assay and standardized ELISA were carried out at the University of Oxford, Competition ELISA at PHE, neutralization assay and whole virion ELISA at Institute for Virology, Philipps University, Marburg, and the NIH ELISA at the NIH.

	ADI 1 : 500 OD	lentivirus IC_50_	competition ELISA	standardized ELISA	NIH ELISA	whole virion ELISA
neutralizing titre	*r* = 0.49 *p* = 0.0064 (**)	*r* = 0.55 *p* = 0.0016 (**)	*r* = 0.21 *p* = 0.26 (n.s.)	*r* = 0.74 *p* < 0.0001 (****)	*r* = 0.3 *p* = 0.11 (n.s.)	*r* = 0.68 *p* < 0.0001 (****)
ADI 1 : 500 OD		*r* = 0.88 *p* < 0.0001 (****)	*r* = 0.38 *p* = 0.037 (*)	*r* = 0.60 *p* = 0.0004 (***)	*r* = 0.21 *p* = 0.26 (n.s.)	*r* = 0.67 *p* < 0.0001 (****)
lentivirus IC_50_			*r* = 0.28 *p* = 0.14 (n.s.)	*r* = 0.74 *p* < 0.0001 (****)	*r* = 0.19 *p* = 0.31 (n.s.)	*r* = 0.74 *p* < 0.0001 (****)
competition ELISA				*r* = 0.18 *p* = 0.35 (n.s.)	*r* = 0.095 *p* = 0.62 (n.s.)	*r* = 0.21 *p* = 0.28 (n.s.)
standardized ELISA					*r* = 0.32 *p* = 0.09 (n.s.)	*r* = 0.89 *p* < 0.0001 (****)
NIH ELISA						*r* = 0.29 *p* = 0.12 (n.s.)

In a pre-clinical NHP model, IgG responses after immunization with AdHu5-based Ebola virus vaccines were measured using an ELISA against GP, where 100% protection against a lethal challenge was predicted by titres of 3700 or greater, while a titre of around 2000 predicted 85% survival. At the time of the 2013–2016 outbreak, this was one of the few measures available to estimate the potential efficacy of candidate vaccines and several Phase I clinical studies used this assay to facilitate comparison [16–21,23]. An interim analysis of the Phase III ring vaccination study of rVSV-ZEBOV in Guinea estimated vaccine efficacy in this trial to be 100% [39]. No immunogenicity data were published for this trial, due to logistical difficulties associated with collecting biological samples, therefore, it is unclear which components of the immune response to vaccination mediated this protective effect. However, Phase I trials of the same vaccine at the same dose, in the USA, induced IgG against Zaire-Mayinga GP with a geometric mean titre (GMT) of 1429 (95% CI: 808–2526) [19] at day 28 post-vaccination, which is probably higher than the level achieved 7–10 days post-vaccination by which time no new cases were observed in the rVSV-ZEBOV ring vaccination trial. A GMT of 1429 is well below the titre required to protect 100% of non-human primates in the experimental model. ChAd3 ZEBOV given at a dose of 1 × 10^11^ viral particles (vp) induced titres comparable to those induced by rVSV-ZEBOV, with day 28 GMTs of 1256 (95% CI: 380–4154) in US adults and 1447 (760–2757) in Malian adults [21]. Peak titres after ChAd3 is boosted with MVA ZEBOV were significantly increased to 11 970 (8752–16 353) [18].

Although the use of different assays (summarized in table 2) limits comparison of immunogenicity across clinical trials, there is still much to be learnt from comparisons of groups within trials. DeSantis and co-workers showed a limited dose effect on anti-GP IgG titres, as assessed through ELISA, when ChAd3 EBOV was tested at 2.5 × 10^10^ vp and 5 × 10^10^ vp. Peak titres and response rates were similar in these two groups both at peak and at day 180, indicating that durability was also comparable [20]. However, in a trial by Ledgerwood *et. al.*, which compared a mixture of ChAd3 ZEBOV and ChAd3 SUDV in a 1 : 1 ratio, each at 1 × 10^10^ or 1 × 10^11^ vp, indicated that a 10-fold higher dose induced significantly higher geometric mean ELISA titres of anti-GP IgG despite similar response rates [17]. In the same trial, 100% of volunteers in the high dose group developed antibody responses against the vaccine strain of Zaire glycoprotein (Mayinga), while only 90% developed responses against glycoprotein from the outbreak strain (Guinea). Additionally, the response rates induced against the vaccine strain Sudan glycoprotein (80% in the high dose group and 70% in the low dose group) were lower than for Zaire (90 and 100%) despite receiving the same dose of each. This may reflect the different sensitivities of the two assays.

The rapid development of multiple vaccine candidates in parallel during the recent outbreak has highlighted the need for standardization of assays that measure humoral immunogenicity. Correlating responses measured by different assays and the inclusion of reference standards can aid comparisons of the quality and quantity of the humoral response induced by different vaccine candidates and support decisions on which to take forward for further development.

### (b) Neutralization assays

There is a lack of discrimination between neutralizing and non-neutralizing antibody levels through ELISA output and while non-neutralizing antibodies play an important role in curbing infection, there is also a concerted effort to identify neutralizing antibodies (NAb) as a key determinant of infection control in many disease settings [43,44]. At present, there are data suggesting that both neutralizing and non-neutralizing antibodies are important in the EVD setting and as such measuring both aspects of humoral immunity through ELISA and neutralization assays will be informative.

Virus neutralization assays (e.g. fluorescent antibody virus neutralization (FAVN) assay or plaque reduction neutralization test (PRNT)) can measure NAb responses. However, there is a prerequisite for highly trained staff and access to containment level 4 facilities to work with *Ebolavirus*, which are not widely available: for example, a single laboratory based in Marburg analysed samples from most of the Phase I studies. Additionally, these assays necessitate careful and considered planning resulting in low-throughput, expensive and time-consuming assays to prevent accidental exposure [45,46]. To circumvent these restrictions, assays using non-pathogenic, replication-defective pseudotyped viruses (PVs) have been developed that facilitate the study of highly pathogenic viruses in standard containment level 2 laboratories. PVs are chimeric virions, which comprise the structural and enzymatic core of one virus with a heterologous envelope protein, e.g. EBOV glycoproteins [47]. Typically, retroviruses (lentiviruses and gammaretroviruses) or rhabdoviruses (vesicular stomatitis virus) are used as pseudotype cores. Transduction of permissive target cells, facilitating genome transfer and subsequently reporter protein expression, allows a quantifiable read-out of transduction and produces a quantitative read-out. However, pre-incubation of PV with NAb which bind an envelope protein, and can inhibit cell entry, will result in a lower level of quantifiable reporter protein expression.

There are many PV assays which have been developed to assess neutralizing antibodies toward the filovirus family members and indeed a number of these assays have been used in a WHO collaborative study to establish a reference standard for antibodies to Ebola virus [41]. The data from this study suggest that PV assays, while offering an obvious improvement to live NAb assays, may generate false positives and as such any new filovirus PV assay requires stringent standardization using validated negative and positive controls.

There have been a limited number of PV assays used in recent clinical trials, which may reflect the urgent need to rank vaccine modalities during the 2013–2016 Ebola outbreak and the concerted effort to screen using live neutralization assays. However, there are a number of informative comparisons between live EBOV neutralization and PV neutralization during some of the more recent clinical trials which will help establish the usefulness and early application of these assays in screening and ranking vaccine candidates (table 2).

In 2010, neutralizing antibodies, as measured with a single-round infection assay with EBOV GP-pseudotyped lentiviruses, were measured following a single IM vaccination in a Phase I clinical trial of AdHu5 vaccine expressing EBOV GP antigen. With the exception of one subject, sera did not inhibit virus entry [14]. The same team also used EBOV or MARV GP-pseudotyped lentiviruses to measure neutralizing antibody activity after 3 or 4 DNA vaccinations. No significant Marburg virus neutralizing activity was observed following the DNA vaccines, and only low-level EBOV neutralizing activity was measured following the fourth DNA vaccination [15]*.*

These data have helped evaluate vaccine regimens and have identified those vaccines and regimens which yield improved neutralizing responses. In a recent Phase I clinical study in Oxford, seven different assays of humoral immunity were undertaken on samples from 30 vaccinees, providing a unique opportunity to assess the relationship between different measures of immunogenicity (table 3). Volunteers were administered a single dose of ChAd3 EBOV GP and a subset were then administered a booster dose of a MVA strain, encoding the same Ebola virus glycoprotein. Two assays were used to measure neutralizing antibodies; direct neutralization of live EBOV (Mayinga strain) and a pseudotyped lentivirus expressing the glycoprotein from the Mayinga strain, with a read-out of 50% inhibitory concentration (IC_50_). Neutralizing antibody titres to live EBOV (Mayinga strain) were low at 28 days after the ChAd3 dose while the levels were significantly higher 14 days after the MVA boost vaccine. Again, low-level inhibition of infection was observed post-prime, using a pseudotyped lentivirus expressing the GP from the Mayinga strain of EBOV, and the levels of NAb increased significantly post-boost. Importantly, neutralizing antibody titres in the live EBOV and PV assay correlated positively with each other (*r* = 0.57, *p* = 0.001) as did titres measured by ELISA and live EBOV (Mayinga strain) neutralization assay (*r* = 0.8, *p* = 0.0001) [18].

NAb were also assayed in vaccinees who received rVSV-ZEBOV, through the use of VSV pseudovirions expressing the glycoprotein from the EBOV 1995 Kikwit strain, and a live neutralization assay with infectious EBOV isolate Mayinga. NAb have been detected in vaccinees receiving as little as 3 × 10^5^ pfu rVSV-ZEBOV, and the two NAb assays showed significant increases in neutralizing antibodies after all doses of rVSV-ZEBOV (ranging from 3 × 10^5^ pfu to 5 × 10^7^ pfu) [22]*.* A strong correlation between antibody titres as assessed with glycoprotein ELISA and VSV pseudovirions or live virus neutralization titres were demonstrated. A significant correlation between vaccine dose and neutralizing antibody titres was also identified using the VSV pseudovirions but such correlations were not observed using whole virions or infectious Ebola virus. This may reflect the lower sensitivity and thus weaker discriminatory capacity of whole virions or infectious Ebola virus assays*.*

The increased interest in measuring NAb post vaccination toward highly pathogenic viruses and the suggested increased sensitivity in established and standardized PV assays will underpin the continued development and use of PV assays for broad-spectrum detection of post-vaccination humoral responses.

## 4. Cellular immune response to vaccination

Baize and co-workers analysed samples from patients in two large outbreaks in 1996 in Gabon and compared cellular and humoral responses between survivors and patients who succumbed to infection to determine the relative contribution of different immune parameters to EVD outcome [7]. Survival was associated with early and rapid rises in IgG directed mainly toward the viral nucleoprotein, in addition to activation of CD8^+^ T cells with upregulation of CD28, FasL, perforin and IFNγ. CD8^+^ T cells specific to the Ebola virus nucleoprotein are also associated with protection in a mouse model of EVD [48] and T cells induced by rAd5 ZEBOV GP vaccination in NHP afford protection in a lethal challenge model [6], while *in vivo* depletion of CD8^+^ T cells using a monoclonal antibody abrogated protection in four out of five NHP, whereas passive transfer of IgG did not induce protection. Therefore, enumeration of antigen-specific T cell responses to candidate Ebola vaccines is an important aspect of the immunological outcomes for studies in humans. Many clinical trials of replication-deficient viral vectored vaccines have reported T cell responses as measured by flow cytometry with intracellular cytokine staining (ICS) using pools of peptides spanning the glycoprotein to stimulate peripheral blood mononuclear cells (PBMC). Comparisons across studies are impeded by differences in sample types used (freshly isolated versus cryopreserved PBMC), variation in denominator used to report responses (memory versus total CD3^+^) and differences in data analysis methodology [17,18,23,49]. Some groups have also reported glycoprotein-specific T cell responses by IFNγ ELISPOT in addition to ICS; however, again differences in responses between fresh and frozen PBMC make broad comparisons difficult.

Although comparisons between all the trials are unreliable, limited comparisons are possible; e.g. when using fresh PBMC with IFNγ ELISPOT, rAdHu5 and ChAd3 vectors encoding EBOV GP induced antigen-specific T cell responses of a similar magnitude [18,24]. For the vaccine regimen where ChAd3 vectors encoding EBOV and SUDV GP were mixed, only the responder frequency and not the magnitude of response was reported; however, responder frequency to the mixture of vectors was similar to that observed to ChAd3 EBOV GP alone.

However, and possibly more importantly, comparisons between groups within the same study have yielded interesting insights into the use of viral vectors that have broader relevance beyond Ebola virus vaccines. For example, a large trial in Oxford comparing MVA-BN Filo priming and AdHu26 ZEBOV boosting demonstrated for the first time in humans that priming with the MVA vector and boosting with an adenovirus is at least as effective at inducing antigen-specific CD8^+^ T cells as the conventional delivery of Ad-prime followed by MVA boost [23].

Surprisingly, the rVSV-ZEBOV vaccine, which to-date has been the only vaccine for which field efficacy has been assessed, has not yet been described for T cell-inducing capability [19,22]. In cynomologous macaques immunized with rVSV-ZEBOV, cytokine-secretion from CD8^+^ T cells stimulated with GP peptides was not detectable after immunization; however, responses were detected in vaccinated animals after EBOV challenge, suggesting that immunization may have primed a response [50]. If T cell responses to the rVSV-ZEBOV have been assessed in Phase I studies, then the absence of CMI would be of interest to the vaccine development field as maintenance of humoral immunity is obviously dependent on CMI, yet the failure to detect such a response peripherally is both surprising and interesting from the immunologist's perspective.

## 5. Achievements of Phase I studies during the 2013–2016 outbreak

As the immediate response to the 2013–2016 EVD outbreak in West Africa begins to wind down, scientists and policy makers involved in vaccine development can reflect on both the progress made by the field in this difficult time as well as implications for future outbreaks. Notable gains and successes have been achieved, including rapid approval and completion of clinical studies for both existing and newly produced candidate EVD vaccines which underpins the capacity of both ethical and regulatory authorities to expedite approvals in the context of public health emergencies. Publication of data from studies undertaken in response to the outbreak has also been rapid with many journals setting aside policies relating to prior disclosure of data to allow data sharing with WHO and other bodies, without prejudice of future publication.

That vaccines against EVD existed at all is partly due to North American biodefense priorities, rather than a planned strategy of preparedness for rapid response to emerging pathogens. The efficacy of the rVSV-ZEBOV vaccine in the Phase III trial in Guinea in the context of a primarily IgG-mediated response suggests that an efficacious EVD vaccine may be comparatively easy to produce, compared with efforts to generate vaccines for diseases such as malaria, HIV and tuberculosis. Indications from the NHP vaccine model with rAd5 EBOV GP and analysis of immune responses in EVD survivors indicated that a CD8^+^ T cell component would be a prerequisite of an effective EVD vaccine. Yet the rVSV-ZEBOV vaccine may not induce a significant CMI component based on pre-clinical data. An additional consideration is that passive transfer of Ebola-specific antibody can fail to protect NHPs from lethal Ebola virus challenge, indicating that antibody titres may be a non-mechanistic correlate of protection and that qualitative aspects of the antibody response and/or cellular immunity that are not readily measured also play key roles in protection [51]. It is likely that the mechanism of protection and protective level of a particular immune component may well differ in an experimental model in NHPs, challenged in a manner that may not accurately reflect natural exposure and in humans exposed to natural infection. This highlights the importance of not being over-reliant on immunological correlates derived from pre-clinical testing. Clearly, different vaccine delivery methods induce qualitatively different immune responses; induction of long-lasting humoral immunity in the absence of T cells seems unlikely, so the ability of prime-boost regimens to induce durable CMI and humoral immunity may be desirable for longevity of protection.

## 6. Addressing future threats

**Figure 1.**
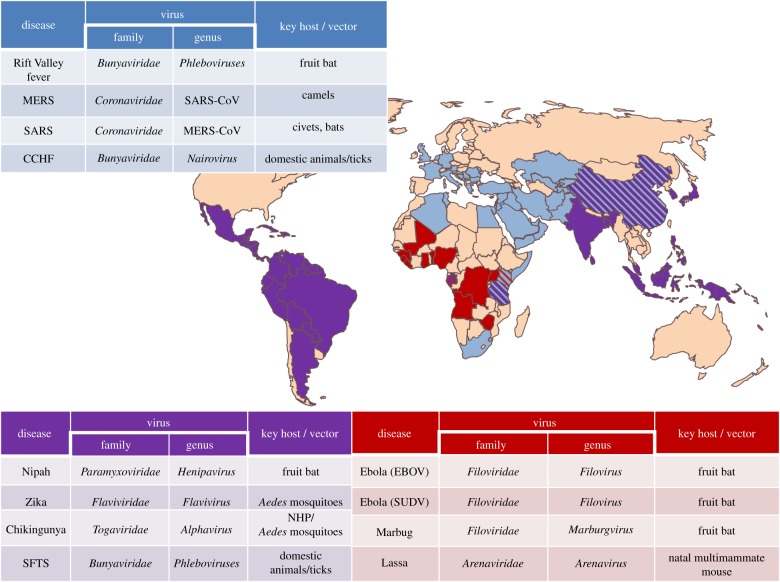
Schematic representation of potential multivalent vaccine strategies targeting WHO priority emerging pathogens. Diseases are broadly grouped according to geographical distribution and incidence. MERS, Middle East respiratory syndrome; SARS, severe acute respiratory syndrome; CCHF, Crimean–Condo haemorrhagic fever; SFTS, severe fever with thrombocytopenia.

In December 2015, WHO held a workshop on the prioritization of pathogens, generating a list of seven diseases requiring urgent R&D, with a further three diseases requiring action as soon as possible [52]. This list encompasses emerging diseases with potential to generate a public health emergency and for which no, or insufficient, preventative or curative solutions exist. Crimean Congo haemorrhagic fever (CCHF), EVD, Marburg haemorrhagic fever, Lassa fever, MERS, SARS, Nipah and Rift Valley fever virus were identified as priorities for emerging pathogen research, with Zika, chikungunya and severe fever with thrombocytopaenia syndrome (SFTS) determined to be serious and necessitating further action as soon as possible. This EVD outbreak has focused attention on emerging pathogens, shifting their place in the public consciousness from a theoretical emergency to a very tangible threat to global health. Vaccine developers must now think in terms of addressing emerging pathogens as an entirety rather than a collection of individual highly virulent pathogens. To this end, the recent outbreak has yielded some valuable insights. Firstly, in the context of an ongoing outbreak, a single dose of a viral vector vaccine could be sufficient to induce at least short-term protective immunity against filoviruses. This can usefully protect front-line healthcare workers and communities, thereby reducing the spread of an epidemic in the earliest stages [53]. Where the outbreak pathogen is readily identified a single dose of a recombinant adenoviral or VSV vector may suffice for outbreak control. However, to counter the threat of infectious outbreaks which may emerge unpredictably, large-scale prophylactic immunization of healthcare workers would be a useful first line of defence. A single-dose multivalent vaccine encoding antigens from several putative emerging pathogens could be particularly valuable in such a scenario. For example, recombinant MVA would be suited to this purpose, given the potential to insert various foreign genes at numerous sites in the genome under a number of promoters [31], in contrast with adenovirus vectors that have a limited cloning capacity to carry foreign genes. Immunity could subsequently be boosted with an adenoviral vector expressing a specific transgene, once the outbreak pathogen is identified. A study of an EVD vaccine regimen employing MVA as a priming vector and AdHu26 as a boost has demonstrated that this is a feasible approach [23]. Speculatively grouping emerging pathogens according to geographical incidence may be one strategy for determining components of multivalent vaccines and an example of a potential strategy for designing vaccines using this approach is suggested in figure 1.

Almost all of the current EVD vaccine candidates use GP as the antigen due to abundant expression on the surface of the virus, and there is extensive pre-clinical data reflecting promising immunogenicity and efficacy against challenge for this target antigen. Studies of patients with EVD in two outbreaks in Gabon revealed that in survivors, IgG responses were largely directed against nucleoprotein [7]. The same observation was made in a small study of EVD cases in the USA [10]. Characterization of immune responses in natural *Ebolavirus* infection have also revealed that exposure can induce cross-reactive antibodies capable of neutralizing multiple *Ebolavirus* species [54]. This study of naturally acquired immunity in survivors of a BDBV outbreak in Uganda in 2007 demonstrated for the first time that potent neutralizing antibodies to the glycan cap of GP could inhibit several *Ebolaviruses*, including SUDV, and protect guinea pigs from a heterologous challenge with EBOV which may offer insight into immunogen design to confer heterosubtypic immunity across multiple *Ebolaviruses*. Given the very large number of survivors of the 2013–2016 outbreak, significant efforts should be made to characterize the immunity that afforded protection in the face of infection with such a highly lethal pathogen, to help inform antigen selection for future vaccines and advance our understanding of post-EVD immunology. Whether vaccines need to emulate these naturally occurring mechanisms remains to be seen, but these observations are of critical importance to help advance the field.

## 7. Novel targets for vaccines against highly lethal pathogens

Closer examination of the priority pathogens highlighted by the WHO may facilitate development of broad-spectrum vaccines; viruses with small genomes that have obvious vaccine candidate antigens or where pre-clinical data are highly indicative [55]. In this setting, data from the One Health approach, which combines human, animal and environmental considerations to address global health challenges, are invaluable; for example, immunogenicity data derived from studies in zoonotic reservoirs can be applied to vaccine development [56,57]. Furthermore, studies of families of viruses using technological advances such as cryo-electron microscopy, B cell cloning and antibody repertoire sequencing may yield novel targets that may be effective for tackling emerging pathogens. Phylogenetically related viruses that share receptor-binding domains, and epitopes within envelope proteins may well share therapeutic and vaccine targets [58]. As discussed above, great efforts have been made to characterize the magnitude of neutralizing antibody titres induced by vaccination, yet the detail of these entry-blocking mechanisms has not yet been fully utilized by vaccinologists. Detailed understanding of host–pathogen interactions has yielded numerous opportunities for vaccines against other diseases, including malaria and HIV [59]. In-depth understanding of the processes associated with inhibition of receptor binding, such as conformational rearrangement of glycoproteins required for viral fusion, could possibly identify ubiquitous targets for mAbs that can prevent infection of multiple virus species [60]: for example, the entry of all filoviruses into mammalian cells utilizes the same endosomal receptor [61], the Niemann-Pick C1 protein, presenting an obvious target for inhibition of virus entry.

## 8. Conclusion

Outbreaks of highly pathogenic diseases such as EVD are devastating for the populations affected and will always focus attention on control strategies to prevent future occurrences. While it is widely acknowledged that the global public health response to the 2013–2016 was tragically slow to begin, immunologists and vaccinologists now have a valuable opportunity to learn as much as possible from this disaster. As well as probing the vaccine-induced responses from the many clinical trials that have taken place, detailed characterization of naturally acquired immunity in both survivors of EVD and asymptomatic, EBOV IgG-seropositive members of communities should be systemically compiled. Previous data from Gabon have illustrated that substantial proportions of rural populations have evidence of EBOV-specific immunity [62]. We now have a collective responsibility to assimilate all the information that this outbreak has generated in order to be best placed when the next epidemic comes, so that we can respond effectively and robustly to curb an outbreak in its infancy.
